# Detection of viral RNA fragments in human iPSC cardiomyocytes following treatment with extracellular vesicles from SARS-CoV-2 coding sequence overexpressing lung epithelial cells

**DOI:** 10.1186/s13287-020-02033-7

**Published:** 2020-11-30

**Authors:** Youjeong Kwon, Sarath Babu Nukala, Shubhi Srivastava, Hiroe Miyamoto, Nur Izzah Ismail, Jordan Jousma, Jalees Rehman, Sang-Bing Ong, Won Hee Lee, Sang-Ging Ong

**Affiliations:** 1 Department of Pharmacology, The University of Illinois College of Medicine, 909 S Wolcott Ave, COMRB 4097, Chicago, IL 60612 USA; 2 Centre for Cardiovascular Genomics and Medicine, Lui Che Woo Institute of Innovative Medicine, Chinese University of Hong Kong (CUHK), Hong Kong, Hong Kong SAR; 3 Hong Kong Hub of Pediatric Excellence, Hong Kong Children’s Hospital, Lab A, 8/F, Tower A, 1 Shing Cheong Road, Kowloon Bay, Kowloon, Kowloon, Hong Kong SAR; 4 Department of Medicine and Therapeutics, Faculty of Medicine, CUHK, Hong Kong, Hong Kong SAR; 5 Division of Cardiology, Department of Medicine, The University of Illinois College of Medicine, 909 S Wolcott Ave, COMRB 4097, Chicago, IL 60612 USA; 6 Department of Basic Medical Sciences, University of Arizona College of Medicine – Phoenix, 425 N 5th St., Bldg ABC1 Rm 426, Phoenix, AZ 85004 USA

**Keywords:** COVID-19, Extracellular vesicles, iPSCs, Stem cells, Cardiomyocytes

## Abstract

Coronavirus disease 2019 (COVID-19) caused by the severe acute respiratory syndrome coronavirus 2 (SARS-CoV-2) is a global pandemic. The prevalence/severity of COVID-19 is higher among patients with cardiovascular risk factors. Despite the expression of angiotensin-converting enzyme 2 (ACE2), a receptor for SARS-CoV-2 infection, in cardiomyocytes, there has been no conclusive evidence of direct viral infection although the presence of viral genome within COVID-19 patients’ hearts has been reported. Here, we overexpressed SARS-CoV-2 genes in A549 lung epithelial cells. We then isolated extracellular vesicles (EVs) and detected the presence of viral RNA within these EVs. We observed that human induced pluripotent stem cell-derived cardiomyocytes (hiPSC-CMs) are receptive to these EVs, and viral genes were detectable in the cardiomyocytes. Accordingly, the uptake of viral RNA-harboring EVs led to an upregulation of inflammation-related genes in hiPSC-CMs. Thus, our findings indicate that SARS-CoV-2 RNA containing EVs represents an indirect route of viral RNA entry into cardiomyocytes.

**Supplementary Information:**

The online version contains supplementary material available at 10.1186/s13287-020-02033-7.

Dear Editor,

Since the initial outbreak in China, coronavirus disease 2019 (COVID-19) which is caused by severe acute respiratory syndrome coronavirus 2 (SARS-CoV-2) has evolved into a global pandemic. While COVID-19 affects both healthy individuals and those with comorbid conditions such as cardiovascular diseases, the severity and risk of adverse outcomes of COVID-19 are especially pronounced in the latter [1]. Furthermore, patients with COVID-19 have also been reported to exhibit increased levels of cardiac biomarkers, suggestive of cardiac injury [2]. However, it remains unclear whether exacerbated cardiac injury seen in COVID-19 patients results directly from viral SARS-CoV-2 infection of the myocardium or indirectly from the complications of COVID-19. Cardiomyocytes express angiotensin-converting enzyme 2 (ACE2), the SARS-CoV-2 binding site [3]. However, there is no evidence of direct viral infection of cardiomyocytes to date clinically, although the presence of myocardial inflammation and viral particles among the interstitial cells of the myocardium has been reported [4] and viral RNA has also been detected in some COVID-19 patients’ hearts [5]. The majority of cells in the body are known to release lipid bilayer membrane vesicles, also known as extracellular vesicles (EVs), that are capable of transferring various genetic materials including viral RNAs to other recipient cells [6, 7]. Therefore, in the present work, we hypothesized that SARS-CoV-2-infected cells such as airway epithelial cells secrete EVs carrying viral genetic material that may be taken up by cardiomyocytes and establish an indirect route of SARS-CoV-2 genetic material transmission.

To test whether the viral RNA of SARS-CoV-2 can be transmitted via EVs into cardiomyocytes without the need for direct infection, we transduced A549 lung epithelial cells with lentivirus encoding selected SARS-CoV-2 proteins [8] (Fig. 1a). A549 cells were chosen as a model cell type since COVID-19 appears to mainly infect respiratory tract cells in patients. SARS-CoV-2 genes encoding for two non-structural proteins (*Nsp1* and *Nsp12*) and two structural proteins (envelope *E* and nucleocapsid *N*) were used for this proof-of-principle study. We opted not to include the spike (*S*) protein, which is required for receptor binding and viral entry, in order to focus on EV-mediated transfer of viral fragments into recipient cardiomyocytes that are independent of S-protein-mediated direct viral entry. The use of lentivirus overexpressing viral subunits also allowed us to distinguish EV-mediated SARS-CoV-2 RNA transfer from canonical virus infection since EV preparations inevitably contain infectious virions due to the overlap in size.

**Fig. 1:**
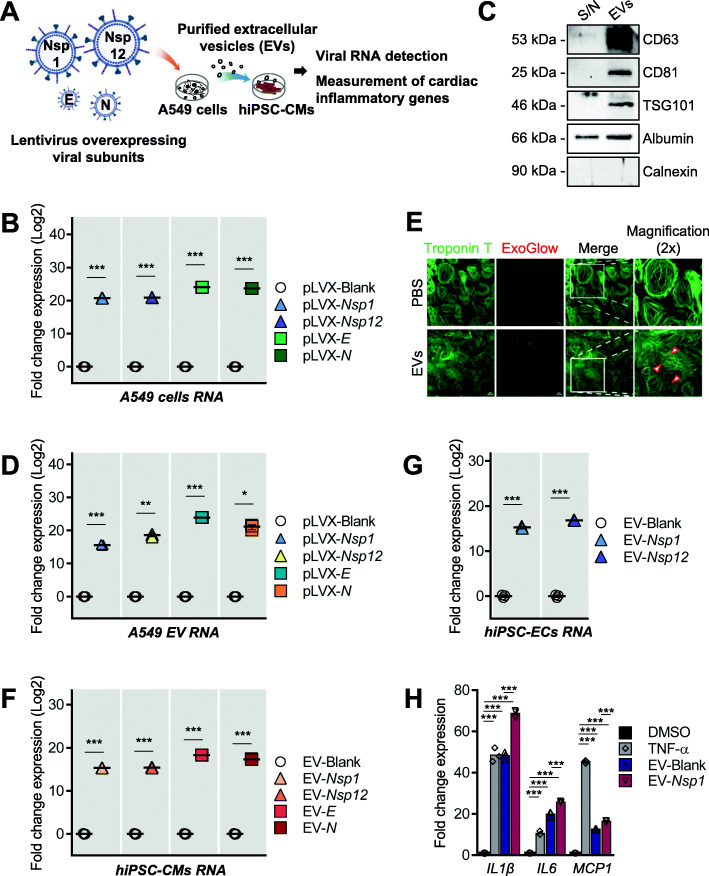
Detection of SARS-CoV-2 synthetic viral RNA fragments in human induced pluripotent stem cell-derived cardiomyocytes (hiPSC-CMs) and endothelial cells (hiPSC-ECs) treated with EVs. **a** Schematic depiction of study design. Nsp1, non-structural protein 1; Nsp12, non-structural protein 12; E, envelope protein; N, nucleocapsid protein. **b** Expression of SARS-Cov-2 genes in A549 lung epithelial cells. A549 cells were infected with indicated lentiviral particles for 48 h and mRNA levels were measured by qRT-PCR (*n* = 3, mean ± S.D). ***P < 0.001 versus pLVX-Blank (Student’s *t* test). **c** Immunoblotting of EV markers demonstrating enrichment in the EV fraction compared to the supernatant. **d** SARS-CoV-2 genetic materials (Nsp1, Nsp12, E, and N) were detected in EVs secreted from A549 lung epithelial cells. EVs were purified from A549 cell culture media, and mRNA levels were measured by qRT-PCR (*n* = 3, mean ± S.D.). *P < 0.05; **P < 0.01; ***P < 0.001 versus pLVX-Blank (Student’s *t* test). **e** Uptake of ExoGlow-labeled EVs (pseudocolored red) or PBS (negative control) by hiPSC-CMs stained with cardiac troponin T (green) was visualized by confocal imaging. Scale bar = 10 μM. Small arrows depict detected EVs within cells. **f** qRT-PCR was performed to detect the presence of viral genes in hiPSC-CMs following EV uptake. (*n* = 3, mean ± S.D.). ***P < 0.001 versus EV-Blank (Student’s *t* test). **g** qRT-PCR was performed to detect the presence of viral genes Nsp1 and Nsp12 in hiPSC-ECs following EV uptake (*n* = 3, mean ± S.D.). ***P < 0.001 versus EV-Blank (Student’s *t* test). **h** Expression of inflammatory genes in hiPSC-CMs. hiPSC-CMs were treated with EVs released by A549 cells transduced with pLVX-Blank or pLVX-Nsp1 lentiviral particles for 6 h and mRNA levels were measured by qRT-PCR (*n* = 3, mean ± S.D.). Tumor necrosis factor-α (TNF-α, 50 ng/ml) was used as a positive control. *** *P* < 0.001 (one-way ANOVA followed by Tukey’s multiple comparisons test). *IL1β*, interleukin 1β; *IL6*, interleukin 6; *MCP1*, monocyte chemoattractant protein 1

Quantitative RT-PCR on total RNA extracted from A549 cells 48 h after lentivirus transduction confirmed the successful overexpression of viral RNAs encoding for Nsp1, Nsp12, E, and N compared to a control empty vector (Fig. 1b). To isolate EVs released by A549 cells, the supernatant of A549 cells grown in a culture medium supplemented with exosome-depleted FBS for 48 h was collected for EV purification. Immunoblotting of EV preparations confirmed the enrichment of the EV markers CD63, CD81, and TSG101 although we did note the presence of albumin most likely due to the PEG-based isolation method used (Fig. 1c). Additional NanoSight analysis of EVs from control, Nsp1, and Nsp12 overexpressing A549 cells confirmed the size of EVs ranged from 50 to 300 nm with no significant difference in terms of particle concentration between all measured groups although there was an increasing trend in the Nsp12 overexpressing A549 cells group (Figure S1).

We next asked whether the RNAs encoding for SARS-CoV-2 are packaged into purified EVs of A549 cells. qRT-PCR revealed the presence of mRNA in purified EVs for each of the four tested SARS-CoV-2 genes (Fig. 1d). We performed a separate validation for Nsp1 and Nsp12 in EVs isolated using a different method based on immuno-magnetic CD63 beads and successfully confirmed the presence of both tested genes in the isolated EVs (Figure S2). To further substantiate the presence of viral genes within EVs, we treated our Nsp1 EV preparation with RNase/protease. As expected, treatment with RNase alone or protease + RNase led to minimal loss of Nsp1, while the addition of detergent led to significant degradation of the enclosed Nsp1 (Figure S3). To study if human cardiomyocytes are able to uptake EVs, we labeled EVs with a fluorescent dye ExoGlow and incubated them (100 μg based on protein quantification) with human induced pluripotent stem cells-derived cardiomyocytes (hiPSC-CMs, 1 × 10^6^ cells). Following 6 h of 37 °C incubation and washout of unbound EVs, we observed the presence of labeled EVs in treated hiPSC-CMs, which was not observed when the cells were incubated with the negative control (PBS without EVs stained with ExoGlow), confirming the successful binding/uptake of EVs by the recipient hiPSC-CMs (Fig. 1e and Figure S4). Moreover, after exposure of hiPSC-CMs to A549 EVs for 24 h, we detected all four tested viral RNAs in the hiPSC-CMs, but we could not detect any significant levels of viral RNAs in hiPSC-CMs treated with control EVs (Fig. 1f). In a separate set of experiments, we exposed hiPSC-CMs to conditioned media collected from Nsp1-overexpressing A549 cells (1 × 10^7^ cells) with and without concurrent treatment of GW4869 (5 μM), an inhibitor of exosome generation. The expression of Nsp1 in hiPSC-CMs was significantly blunted when GW4869 was present, consistent with the involvement of EVs in RNA transfer (Figure S5). Next, we tested whether the phenomenon of uptake of viral gene-containing EVs was also present in hiPSC-derived endothelial cells (hiPSC-ECs) as an additional form of validation. Similar to the hiPSC-CMs, we detected viral RNAs in hiPSC-ECs after A549 EV treatment but not control EVs in a concentration-dependent manner (Fig. 1g, Figure S6). We then assessed whether hiPSC-CM exposure to EVs from A549 cells expressing viral RNA increased inflammatory gene expression. It is known that A549 EVs themselves can increase inflammation [9]. We noted that EVs containing Nsp1 further increased the expression of the pro-inflammatory genes *IL1β*, *IL6*, and *MCP1*, suggesting the transferred viral gene may promote inflammation (Fig. 1h).

There are several limitations to our study. Although the precipitation method used in our study for isolating EVs leads to high recovery, it is associated with the recovery of non-EV components such as proteins. However, our additional experiments including immuno-magnetic isolation of EVs, RNase/protease treatment, and GW4869 treatment support the enrichment of viral genes within EVs. The ExoGlow dye used cannot fully distinguish between binding and internalization of EVs. It should also be noted that the viral genes used in our study are codon-optimized fragments and expressed at a supraphysiological level. Further experiments are needed to validate if EVs are capable of transferring actual viral RNAs or virions at a physiological level and if the transferred RNAs are biologically active. Lastly, while we have only explored hiPSC-CMs and hiPSC-ECs in this study which both demonstrated uptake of EVs containing viral RNA fragments, we were unable to include a negative control cell type. Overall, our results collectively demonstrated that lung epithelial cells expressing SARS-CoV-2 genes can secrete EVs containing viral RNA fragments that can be detected in cardiomyocytes suggesting an indirect route of viral RNA delivery into cardiac cells via EVs. Transfer of viral RNA via EVs should be considered when studying the widespread multi-organ effects of a SARS-CoV-2 infection that has been reported [10], because it indicates that cells which do not express the SARS-CoV-2 receptor ACE2 might still be vulnerable via the uptake of EVs. Further work is needed to clarify whether the entry of SARS-CoV-2 RNA via EVs is sufficient to induce cell injury and inflammation.

## Supplementary Information

**Additional file 1.** Supplementary methods, table, and figures.

## Data Availability

The datasets used and/or analyzed during the current study are available from the corresponding authors on reasonable request.

## Abbreviations

COVID-19: Coronavirus disease 2019
SARS-CoV-2: Severe acute respiratory syndrome coronavirus 2
ACE2: Angiotensin-converting enzyme 2
EVs: Extracellular vesicles
hiPSCs: Human induced pluripotent stem cells
hiPSC-CMs: Human induced pluripotent stem cell-derived cardiomyocytes
IL1β: Interleukin 1β
IL6: Interleukin 6
MCP1: Monocyte chemoattractant protein 1
