# Supplementary material for: Detection of viral RNA fragments in human iPSC cardiomyocytes following treatment with extracellular vesicles from SARS-CoV-2 coding sequence overexpressing lung epithelial cells
Source: Stem Cell Res Ther. 2020 Nov 30;11:514. doi: 10.1186/s13287-020-02033-7 (PMC7703503; doi:10.1186/s13287-020-02033-7)
Supplement: Supplementary file 1 — Additional file 1. Supplementary methods, table, and figures. [file 13287_2020_2033_MOESM1_ESM.docx]

**Detection of Viral RNA Fragments in Human iPSC-Cardiomyocytes following Treatment with Extracellular Vesicles from SARS-CoV-2 Coding-Sequence-Overexpressing Lung Epithelial Cells**

**SUPPLEMENTAL MATERIALS**

Youjeong Kwon, Sarath Babu Nukala, Shubhi Srivastava, Hiroe Miyamoto, Nur Izzah Ismail, Jordan Jousma, Jalees Rehman, Sang-Bing Ong, Won Hee Lee, Sang-Ging Ong

**Address correspondence to**: Sang-Bing Ong, PhD, Centre for Cardiovascular Genomics and Medicine, Lui Che Woo Institute of Innovative Medicine, Chinese University of Hong Kong (CUHK), Hong Kong SAR. E-mail: [sangbingong@cuhk.edu.hk](mailto:sangbingong@cuhk.edu.hk) or Won Hee Lee, PhD, 425 N 5^th^ St, Bldg ABC1 Rm 426, Phoenix, AZ 85004, USA. Email: [whlee@email.arizona.edu](mailto:whlee@email.arizona.edu) or Sang-Ging Ong, PhD, 909 S Wolcott Ave, COMRB 4097, Chicago, IL 60612, USA. E-mail: [sangging@uic.edu](mailto:sangging@uic.edu)

**SUPPLEMENTAL METHODS**

**Cell Culture**

HEK293T cells were purchased from TakaraBio and cultured in Dulbecco’s modified Eagle’s medium (DMEM) with high glucose (Thermo Fisher, 11995-065) supplemented with 10% serum. A549 cells were purchased from ATCC and cultured in Ham’s F-12K medium (Thermo Fisher, 21127022). Human induced pluripotent stem cells-derived cardiomyocytes (hiPSC-CMs) were differentiated using a chemically defined monolayer differentiation protocol as previously described^1^. Briefly, iPSCs at 90% confluence were incubated with differentiation basal medium comprising RPMI 1640 medium (Thermo Fisher, 11875095) and B27 supplement minus insulin (Thermo Fisher, A1895601). CHIR99021 (8 μM) was added to the differentiation basal medium. On day 2, medium was removed and replaced with differentiation basal medium minus CHIR99021. On day 3, the Wnt antagonist, IWR-1, was added to the medium. After 48 hours, medium was removed and replaced with differentiation basal medium without any inhibitors. On day 7, the cells were incubated with complete CM medium consisting of RPMI 1640 medium and B27 supplement plus insulin (Thermo Fisher, 17504044). The medium was changed every 2 days. Monolayers of hiPSC-CMs were cultured for 30 days and subsequently dissociated for experimental use using TrypLE Express (Life Technologies). For measurement of inflammatory genes, hiPSC-CMs were exposed to either TNF-α (50 ng/mL) or EVs (control vs Nsp1) for 6 hours prior to RNA extraction. Endothelial differentiation was performed by using previously described protocols^2,3^. For lateral mesoderm induction, the hiPSCs were split in a 1:12 ratio using EDTA, and the medium was changed to N2B27 medium consisting of 1:1 mixture of DMEM:F12 (1:1) (Thermo Fisher, 11320) and Neurobasal media (Thermo Fisher, 21103) supplemented with N-2 (Thermo Fisher, 17502) and B27 minus vitamin A (Thermo Fisher, 12587) and 6-8 μM CHIR-99021 next day and incubated for 3 days. For endothelial cell induction, cells were cultured in StemPro-34 SFM (Thermo Fisher, 10639011) supplemented with VEGF and forskolin for 3 days. At day 6 post-differentiation, cells were sorted using the human CD144 (VE-Cadherin) MicroBeads and magnetic cell sorting (MACS) system (Miltenyi Biotech, 130-097-857) following the manufacturer’s instructions, and expanded on fibronectin coated plates in EGM2 media (Lonza, CC-3162). The medium was changed every 2 days. All procedures conformed to the UIC institutional review board-approved protocol.

**Production of lentivirus expressing SARS-CoV-2 subunits**

Plasmids encoding for codon-optimized Nsp1, Nsp12, E and N of SARS-CoV-2 with a 2xStrep tag at the C-terminus were kindly provided by Dr. Nevan Krogan^4^. Empty vector or respective overexpression plasmids were packaged into virus using HEK293T cells as the packaging cell line in 10 cm dishes. Target DNA, helper plasmids VSVG and PAX2 were transfected at 9, 3 and 9 μg respectively using Lipofectamine 2000 (Thermo Fisher, 11668027). Infectious supernatant was collected at 48 and 72 hours after transfection and filtered to remove cell debris. Supernatant was then concentrated using Lenti-X Concentrator (TakaraBio, 631232) according to the manufacturer’s protocol. A549 cells were then transduced with either control or overexpression lentivirus with polybrene (8 µg/ml) overnight, and fresh medium supplemented with exosome-depleted FBS (Thermo Fisher, A2720803) was added and incubated for 48 hours before being collected for EV isolation.

**RNA extraction and quantitative real-time reverse transcription-polymerase chain reaction (qRT-PCR)**

Total RNA was isolated using Direct-Zol RNA Miniprep Kit (Zymo Research, R2051). Reverse transcription was performed using the High-Capacity cDNA Reverse Transcription Kit with RNase Inhibitor (Thermo Fisher, 4374966) and qRT-PCR was performed using the PowerUp SYBR Green Master Mix (Thermo Fisher, A25742) on a QuantStudio 7 Flex real-time PCR detector (Thermo Fisher). Relative mRNA levels were normalized to those of GAPDH mRNA in each reaction and undetermined raw Ct values were set to 40 for analysis purposes. Primers sequences are shown in **Table 1**. Three to four replicates per group were used for qRT-PCR.

**Protein extraction and western blot analysis**

EV samples were prepared in 1x RIPA buffer (Sigma, R0278) supplemented with protease and phosphatase inhibitor (Thermo Fisher, 78440). Samples were subjected to electrophoresis on 4-12% NuPAGE Bis–Tris gels (Thermo Fisher, NP0335BOX) and proteins were transferred to nitrocellulose membranes using wet-based transfer system (Bio-Rad). Membranes were incubated overnight with the indicated primary antibodies, followed by incubation for 1 hour with horseradish peroxidase-conjugated secondary antibodies (Cell Signaling; 7074 and 7076). Signals were detected by chemiluminescence. Primary antibodies used include CD63 (Thermo Fisher, 10628D), CD81 (Thermo Fisher, 10630D), TSG101 (Thermo Fisher, MA123296), calnexin (Thermo Fisher, MA3027) and albumin (Proteintech, 16475-1-AP).

**Isolation of extracellular vesicles**

Supernatant of A549 cells was collected at 48 hours after transduction with lentivirus for isolation of EVs. Briefly, supernatant was first centrifuged at 300 x g for 5 minutes, followed by 1500 x g for 10 minutes, filtered (0.2 μM) and concentrated using Ultracel-100K (Millipore). EVs in concentrate were then isolated using Total Exosome Isolation Reagent (Thermo Fisher, 4478359) according to the manufacturer’s instructions overnight at 4°C, followed by centrifugation at 12 000 x g at 4°C for 1 hour. Isolated EVs were immunoblotted for EV markers CD63 and CD81. For immuno-magnetic isolation of CD63-positive EVs, the exosome-human CD63 isolation/detection reagent (Thermo Fisher, 10606D) was used based on the manufacturer’s protocol. EVs were pre-enriched using the Total Exosome Isolation Reagent as described above. For GW4869 (Sigma, D1692) experiments, supernatant was concentrated 100 times using Ultracel-100K following treatment of A549 cells for 48 hours with and without GW4869 (5 μM) before being added to hiPSC-CMs (1 x 10^6^ cells seeded in 1 mL media).

**NanoSight analyses of EVs**

The size distribution and concentration of isolated EVs were measured using NanoSight NS300 (Malvern Panalytical). Samples were processed in triplicates and one mL of EVs diluted 500-fold with PBS was used. Samples were recorded with a camera level set at 14, and detection threshold from 3 to 7. The captured videos were analyzed using the NTA v3.4 software to process size and concentration measurement.

**RNase/protease treatment of EVs**

An exosomal preparation was divided into four equal fractions for the following treatments: i) untreated; ii) RNase only, iii) RNase with proteinase K and iv) RNase, proteinase K and Triton X-100. First, 1% Triton X-100 or an equivalent volume of PBS was added and incubated on ice for 15 minutes. This was followed by the addition of proteinase K (0.05 μg/μl) at 37°C for 20 minutes, after which the reaction was stopped by adding 1x protease/phosphatase inhibitor cocktail. Finally, RNase A (0.5 μg/μl) is added at 37°C for 20 minutes. Samples were then immediately subjected to RNA isolation and qRT-PCR.

**Uptake of EVs by hiPSC-CMs**

Purified EVs were labeled using the ExoGlow^TM^-Protein EV Labeling Kit (System Bio, EXOGP400A-1) which labels internal EV proteins based on the manufacturer’s description. Briefly, 150 μg of EVs were resuspended in 500 μl of PBS and incubated with 1 μl of ExoGlow dye with shaking at 37°C for 20 minutes. For negative control staining, 500 ul of PBS + 1 µl of ExoGlow dye was used. Unincorporated dye was removed by the use of Exosome Spin Columns (Thermo Fisher, 4484449). Labeled EVs were then precipitated overnight as described above and added to hiPSC-CMs for 6 hours at 37ºC. hiPSC-CMs were then fixed and stained with cardiac troponin T (Abcam, AB45932) and imaged by confocal microscopy to visualize uptake of labeled EVs.

**Statistics**

The values presented are the means ± standard deviation from at least three samples. Statistical differences were determined by a 2-tailed, unpaired Student t-test or one-way ANOVA with Tukey’s multiple comparison test as appropriate. A value of P<0.05 was considered significant.

**Table 1. List of primers used in this study**

| **Gene** | **Primer (Forward)** | **Primer (Reverse)** |
| --- | --- | --- |
| *Nsp1* | TTATGGAGCCGACCTCAAATC | GAGTAACGCCACTGCTATGT |
| *Nsp12* | GACGACGCTGTAGTATGCTTTA | TCAGTCTCGGTCCAACATTTC |
| *E* | GTATTTCTCCTCGTCACACTGG | CACCCTGCTGTAAACGTAGAA |
| *N* | CGGGACATGGCTCACTTATAC | GGTGGGTGGAAACGTCTTAT |
| *GAPDH* | GGTGTGAACCATGAGAAGTATGA | GAGTCCTTCCACGATACCAAAG |
| *IL1β* | CCACAGACCTTCCAGGAGAATG | GTGCAGTTCAGTGATCGTACAGG |
| *IL6* | AGACAGCCACTCACCTCTTCAG | TTCTGCCAGTGCCTCTTTGCTG |
| *MCP1* | AGAATCACCAGCAGCAAGTGTCC | TCCTGAACCCACTTCTGCTTGG |

**SUPPLEMENTAL FIGURES**

**
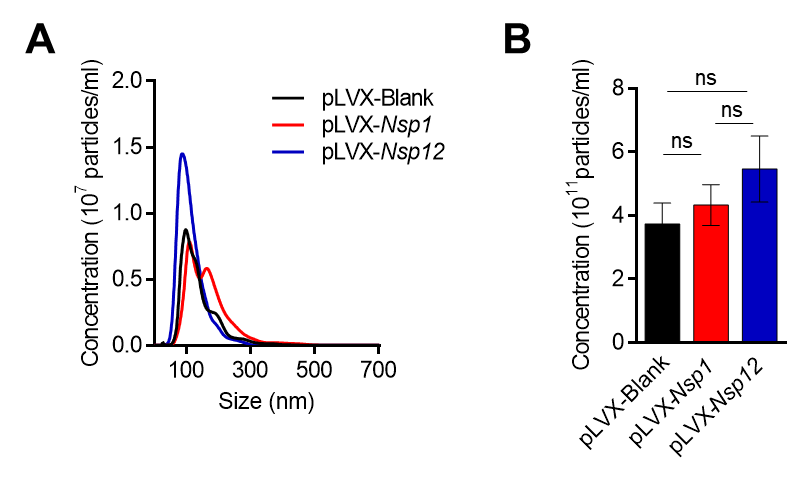
**

**Supplemental Figure 1**. Size measurement and quantification of EVs from A549 lung epithelial cells transduced with pLVX-Blank, pLVX-Nsp1 or pLVX-Nsp12 lentiviruses. **A**. Line histograms depicting the size distribution of isolated EVs as measured by the NanoSight instrument. The mean size of EVs were 139.8 ± 1.9 nm, 166.6 ± 2.4 nm, and 114.1 ± 1.4 nm for pLVX-Blank, pLVX-Nsp1 and pLVX-Nsp12, respectively. **B**, Quantification of NanoSight data showing the concentration of EVs for each group. EVs were collected from 10 mL of culture media in 10 cm dish of A549 cells cultured for 48 hours. The purified EVs were then diluted 500-fold for measurement by the NanoSight instrument (n = 3, mean ± S.D.). ns, not significant (One-way ANOVA followed by Tukey's multiple comparisons test).

**
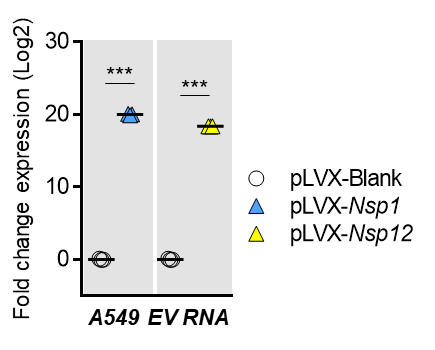
**

**Supplemental Figure 2**. SARS-CoV-2 synthetic genes (Nsp1 and Nsp12) were detected in EVs released from A549 lung epithelial cells. EVs were isolated using immuno-magnetic anti-CD63 beads as an additional verification method. mRNA levels were measured by qRT-PCR (n = 3, mean ± S.D.). ***P<0.001 versus pLVX-Blank (Student’s t-test).

**
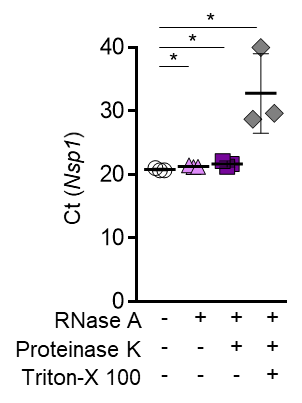
**

**Supplemental Figure 3**. RNase A and Proteinase K sensitivity of Nsp1 within EVs. Nsp1 EVs were purified from A549 cell culture media and treated with either RNase A alone, RNase A and Proteinase K, or RNase A and Proteinase K in the presence of detergent. mRNA levels were measured by qRT-PCR (n = 3, mean ± S.D.). *P<0.05 versus untreated EVs (Student’s t-test).


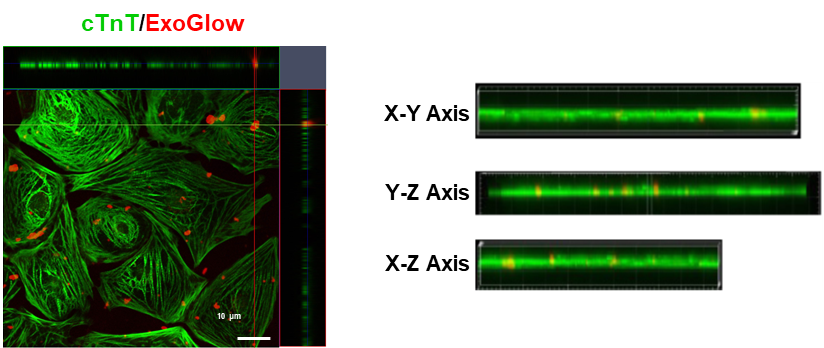


**Supplemental Figure 4**. Visualization of ExoGlow-labeled exosomes (red) added to hiPSC-CMs which were stained with cardiac troponin T (green) by confocal imaging. Orthogonal planes (xy, yz and xz) of the confocal microscope images are depicted on the right. Scale bar = 10 μM.


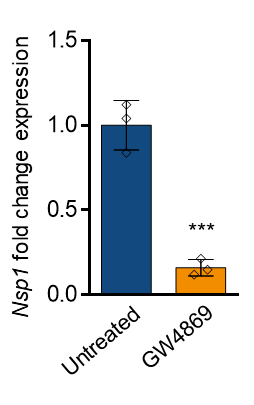


**Supplemental Figure 5**. hiPSC-CMs were exposed to conditioned media collected from Nsp1-overexpressing A549 cells treated with or without GW4869 (5 μM). mRNA levels were measured by qRT-PCR (n = 3, mean ± S.D.). ***P<0.001 versus untreated (Student’s t-test).


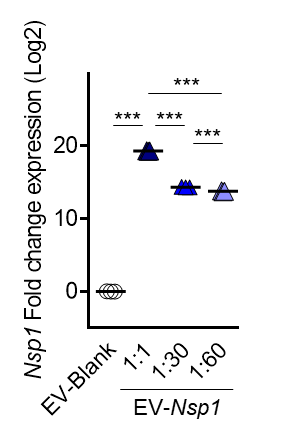


**Supplemental Figure 6**. qRT-PCR was performed to detect the presence of Nsp1 in hiPSC-ECs exposed to serial dilution of Nsp1 EVs or control EVs as indicated (n = 3, mean ± S.D.). ***P<0.001 versus initial dilution (One-way ANOVA followed by Tukey's multiple comparisons test).

1 Kodo, K. *et al.* iPSC-derived cardiomyocytes reveal abnormal TGF-beta signalling in left ventricular non-compaction cardiomyopathy. *Nat Cell Biol* **18**, 1031-1042, doi:10.1038/ncb3411 (2016).

2 Patsch, C. *et al.* Generation of vascular endothelial and smooth muscle cells from human pluripotent stem cells. *Nat Cell Biol* **17**, 994-1003, doi:10.1038/ncb3205 (2015).

3 Ong, S. B. *et al.* Calpain Inhibition Restores Autophagy and Prevents Mitochondrial Fragmentation in a Human iPSC Model of Diabetic Endotheliopathy. *Stem Cell Reports* **12**, 597-610, doi:10.1016/j.stemcr.2019.01.017 (2019).

4 Gordon, D. E. *et al.* A SARS-CoV-2 protein interaction map reveals targets for drug repurposing. *Nature*, doi:10.1038/s41586-020-2286-9 (2020).
